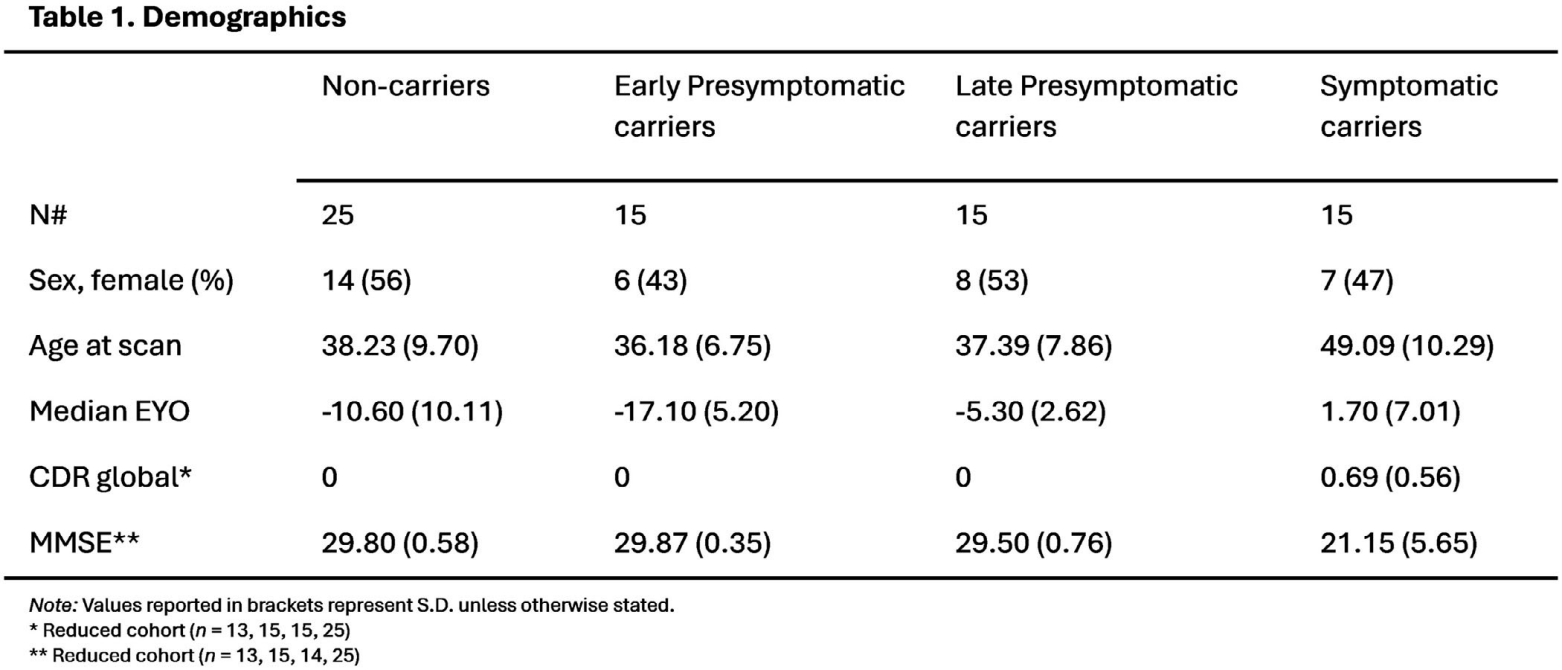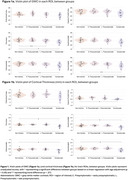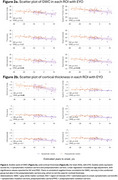# Grey‐White matter contrast as an early brain pathology marker in Familial Alzheimer's disease

**DOI:** 10.1002/alz70856_104802

**Published:** 2026-01-07

**Authors:** Lloyd Prosser, Ian B. Malone, Antoinette O'Connor, Damien Ferguson, Duncan Alston, Helen Rice, Leonard Pieperhoff, Frederik Barkhof, Nick C Fox, Tiago Gil Oliveira, Philip SJ Weston

**Affiliations:** ^1^ Dementia Research Centre, UCL Queen Square Institute of Neurology, London, United Kingdom; ^2^ School of Medicine, University of Dublin, Trinity College, Dublin, Ireland; ^3^ St. James's Hospital, Dublin, Ireland; ^4^ Amsterdam University Medical Center (Amsterdam UMC), Amsterdam, North Holland, Netherlands; ^5^ Department of Radiology and Nuclear Medicine, Amsterdam UMC, Vrije Universiteit, Amsterdam, Netherlands; ^6^ Department of Computer Science and Centre for Medical Image Computing, University College London, London, United Kingdom; ^7^ UK Dementia Research Institute, Queen Square Institute of Neurology, University College London, London, United Kingdom; ^8^ School of Medicine, Institute of Life and Health Sciences (ICVS), University of Minho, Braga, Portugal; ^9^ UK Dementia Research Institute at UCL, London, United Kingdom

## Abstract

**Background:**

Previous work has suggested that the grey‐white matter contrast (GWC) on T_1_‐weighted MRI may be an early marker of neurodegeneration in Alzheimer's disease (AD). Here we aimed to calculate regional GWC in a familial AD (FAD) population and examine its utility in detecting early neurodegeneration in comparison to traditionally used cortical thickness measures.

**Method:**

Seventy individuals from families affected by FAD were included, including presymptomatic mutation carriers, symptomatic mutation carriers and their non‐carrier sibling controls (Table 1). The presymptomatic group was split at median estimated years to onset (EYO; *median* = ‐9.5 yrs), into *early‐presymptomatic* and *late‐presymptomatic*. Both GWC and cortical thickness were estimated using Freesurfer for six pre‐defined regions of interest (ROIs), known to be particularly vulnerable to AD pathology: entorhinal cortex, superior frontal lobe, precuneus, superior parietal lobe, inferior parietal lobe, and supramarginal gyrus. All analyses were age adjusted.

**Result:**

Symptomatic mutation carriers had lower GWC than non‐carriers in ROIs: the precuneus and superior parietal lobe (*p* < 0.05), and inferior parietal lobe and supramarginal gyrus (*p* < 0.07), while cortical thickness was lower in symptomatic carriers across five ROIs (Figure 1). In presymptomatic mutation carriers, GWC was significantly correlated with EYO (a proxy measure of disease stage). Lower GWC was associated with closer proximity to symptom onset in both the inferior parietal and supramarginal cortices, with a negative trend (*p* ≤ 0.1) for all but one of the ROIs (Figure 2a). This association with EYO during the presymptomatic period was not consistently present for cortical thickness, with only one ROI showing any trend towards an association (Figure 2b).

**Conclusion:**

GWC is associated with disease stage and proximity to onset during the 20 years prior to symptoms manifesting; a crucial window for AD therapeutic trials. While cortical thickness measures can detect atrophy in the symptomatic disease stage of AD, it appears less able than GWC to track changes during presymptomatic stages. While the influence of factors such as age and amyloid related inflammation require further investigation, GWC shows promise as an easily accessible clinical MRI tool for disease staging and tracking during the years preceding clinical onset.